# Intestinal necrosis in a patient with Behçet's disease and infective endocarditis: a case report

**DOI:** 10.3389/fcvm.2026.1911451

**Published:** 2026-07-31

**Authors:** Dan Wang, Xia Han, Haibo Ren

**Affiliations:** Department of Critical Care Medicine, Wuhan Asia Heart Hospital, Wuhan, China

**Keywords:** Behçet's disease, extracorporeal membrane oxygenation, infective endocarditis, intra-aortic balloon pump, mesenteric ischemia, venous thrombosis

## Abstract

Vascular Behçet's disease (BD) complicated by infective endocarditis (IE) presents extreme challenges during cardiac surgery and postoperative care. We report a fatal case in which a patient with vascular BD, after complex cardiac surgery for IE, developed extensive mesenteric arteriovenous thrombosis while on intra-aortic balloon pump (IABP) and extracorporeal membrane oxygenation (ECMO) support. This rapidly progressed to intestinal necrosis and perforation. This case highlights the heightened, and likely underrecognized, risk of catastrophic mesenteric thrombosis when mechanical circulatory support is employed in patients with vascular BD.

## Introduction

Behçet's disease (BD) is a systemic vasculitis of unknown etiology that can affect both the arterial and venous systems, leading to thrombosis, aneurysm formation, and vascular stenosis (1). In vascular BD, which is characterized by severe vascular involvement, cardiac involvement is rare but may manifest as valvular lesions, coronary arteritis, and intracardiac thrombosis (2, 3). Due to the underlying vasculitis, the mechanisms of thrombotic events in BD patients—characterized by increased venous susceptibility and a mutually reinforcing relationship between thrombosis and inflammatory responses—differ significantly from those in the general population (4). The incidence of BD complicated by infective endocarditis (IE) is low; however, owing to fragile vessel walls and a systemic hypercoagulable state, the surgical risk and the rate of postoperative complications are markedly elevated (5).

Intra-aortic balloon pump (IABP) and extracorporeal membrane oxygenation (ECMO) are indispensable mechanical circulatory support modalities in the management of cardiogenic shock. Among the complications associated with mechanical circulatory support, lower limb ischemia is relatively common (6). Although mesenteric ischemia is less frequently reported, once it occurs, the mortality rate is extremely high (7). IABP/ECMO-related mesenteric ischemia is mostly caused by arterial embolism or nonocclusive mesenteric ischemia, while cases resulting from venous thrombosis are exceedingly rare.

This article reports a case of vascular BD complicated by IE, in which the patient developed extensive mesenteric arteriovenous thrombosis leading to intestinal necrosis during postoperative support with an IABP and ECMO after complex cardiac surgery. Through a review of the literature, we examine possible mechanisms contributing to mesenteric ischemia in this context to aid clinical detection and prevention.

## Case report

**Figure 1:**
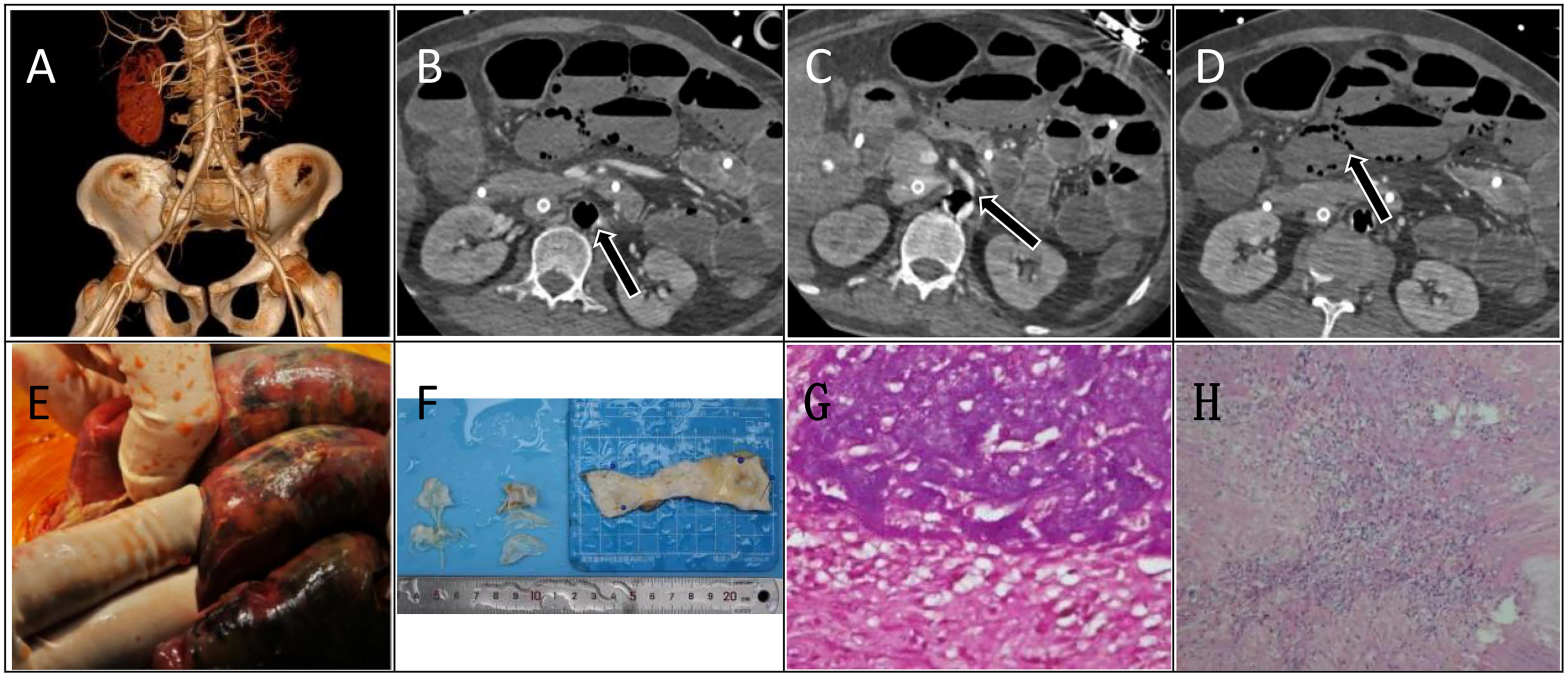
Imaging, intraoperative findings, surgical specimens, and histopathological examination of the patient. (A) Preoperative CTA showing multiple ulcerative lesions in the descending aorta. (B) After IABP balloon inflation, the balloon fully fills the abdominal aorta (black arrow) and compresses the origin of the superior mesenteric artery **(C)**, black arrow). (D) CT shows dilated bowel loops with multiple air-fluid levels, gas in the superior mesenteric vein and its branches, pneumatosis intestinalis of the small bowel wall, and free gas in the mesenteric region (black arrow). (E) Exploratory laparotomy reveals extensive small intestinal necrosis with a gray-white and black-brown mottled appearance, accompanied by multiple perforations, widespread mesenteric venous thrombosis, and active bleeding in some mesenteric arteries. (F) Postoperative gross specimen, from left to right, gross specimens of the mitral valve, aortic valve, and aortic wall. **(**G,H) Pathological examination (staining, magnification × 100).

A 47-year-old man was admitted with a history of chest tightness and shortness of breath upon exertion for over 40 days, which had worsened over the previous 10 days. He had been experiencing intermittent chest tightness and shortness of breath on exertion since late September 2025, which could be relieved by rest. On 3 October 2025, an echocardiogram at another hospital revealed “prolapse of the left coronary cusp of the aortic valve with moderate-to-severe regurgitation, left ventricular enlargement, and ascending aortic dilation”. In early November, the symptoms worsened with the onset of orthopnea and bilateral lower limb edema. A follow-up echocardiogram revealed: “Aortic perivalvular abscess ruptured into the left ventricle, with very severe regurgitation on the left ventricular outflow tract side; global cardiac enlargement; severe tricuspid regurgitation; severe pulmonary hypertension; moderate mitral regurgitation; a patent foramen ovale (left-to-right shunt); reduced right ventricular systolic function; and a small amount of pericardial effusion”. The patient had a long history of recurrent oral ulcers over many years and a history of tooth extraction three months prior. Upon admission, computed tomography angiography (CTA) of the thoracic and abdominal aorta showed multiple ulcers in the descending aorta (Figure 1A), with no significant stenosis observed in the celiac trunk, superior mesenteric artery, or inferior mesenteric artery.

Given the progressive worsening of the patient's heart failure, an emergency operation was performed under general anesthesia, moderate hypothermia, and cardiopulmonary bypass on 24 November 2025. Intraoperative exploration revealed an infection and avulsion at the junction of the left and right coronary cusps of the aortic valve. This had formed a pseudoaneurysm with vegetations measuring approximately 3–4 cm, which had also involved the anterior mitral leaflet. The surgical procedures performed included aortic root replacement with a valved conduit (Bentall procedure), skirted double annulus enlargement, mechanical mitral valve replacement, tricuspid annuloplasty, vegetation debridement, left atrial appendage thrombus removal, left atrial appendage closure, and intraoperative pacemaker implantation. Intraoperatively, due to difficulty in weaning from cardiopulmonary bypass, IABP and ECMO were sequentially placed for support. Delayed sternal closure was performed due to myocardial edema and high chest tube output.

Following surgery, the patient was transferred to the intensive care unit and maintained on ECMO, IABP, and high-dose vasoactive drugs. Systemic anticoagulation was not administered in the early postoperative period because of high chest tube output, coagulopathy, and delayed sternal closure. Follow-up echocardiography on 28 November 2025 revealed improvement in myocardial edema and a decrease in chest tube output, and sternal closure was performed on the same day. Following sternal closure, weaning from ECMO was attempted but failed due to hemodynamic instability, with the left ventricular ejection fraction dropping to 30%. Anticoagulation was initiated after bleeding stabilized, given the ongoing high thrombotic risk associated with the mechanical valve, ECMO circuit, and IABP catheter. On 30 November 2025, the patient developed significant abdominal pain and rigidity. Emergency abdominal CT revealed that the IABP balloon, when inflated, filled the abdominal aorta (Figure 1B) and compressed the origin of the superior mesenteric artery (Figure 1C); dilated bowel loops with multiple air-fluid levels, gas in the superior mesenteric vein and its branches, pneumatosis intestinalis of the small bowel wall, and free gas in the mesenteric region were also noted (Figure 1D). Acute mesenteric ischemia was diagnosed, and the IABP was immediately removed. On 2 December 2025, the patient's hemodynamics fluctuated again, the abdominal condition worsened, and bowel sounds disappeared. D-dimer and blood lactate levels continued to rise, and inflammatory markers increased sharply. Emergency exploratory laparotomy was performed. The laparotomy revealed extensive small intestinal necrosis with a gray-white and black-brown mottled bowel wall, accompanied by multiple perforations, widespread thrombosis of the mesenteric veins, and active bleeding in some mesenteric arteries (Figure 1E). Due to the extensive extent of necrosis precluding bowel resection, only exploratory laparotomy with closure was performed. Postoperative specimens and pathology are shown in Figures 1F–H. Figure 1F shows, from left to right, gross specimens of the mitral valve, aortic valve, and aortic wall. Figure 1G presents H&E and Gram staining of the aortic valve tissue: H&E staining reveals local endocardial disruption with vegetations and inflammatory granulation tissue infiltrated by neutrophils and lymphocytes, while Gram staining demonstrates clustered Gram-positive cocci. It is important to note that, despite the intraoperative findings and postoperative pathology being highly suggestive of infective endocarditis, multiple blood cultures obtained both before and after surgery remained negative. Therefore, the definitive diagnosis of infective endocarditis in this case was not based on blood culture evidence, but rather on the direct intraoperative visualization of characteristic destructive lesions (valvular avulsion, pseudoaneurysm, and vegetations) and postoperative histopathological examination (HE staining confirming inflammatory granulation tissue and valve destruction, with Gram staining only demonstrating morphologically positive cocci). Figure 1H shows H&E and Elastic van Gieson (EVG) staining of the aortic wall. H&E staining demonstrates neointima formation and atherosclerotic plaques, accumulation of mucopolysaccharide-like material in the medial layer, disorganized smooth muscle cells with focal necrosis, and multifocal inflammatory cell infiltration (predominantly neutrophils and lymphocytes). The adventitia shows interstitial fibrosis and thickened vasa vasorum with inflammatory infiltration. EVG staining reveals focal disruption, rarefaction, and irregular arrangement of elastic fibers in the media. Despite maximal life support postoperatively, septic shock and multiple organ failure were irreversible. The patient died on 3 December 2025 (postoperative day 10). A chronological summary of the major clinical events is provided in Table 1.

**Table 1:** Timeline of Major clinical events and anticoagulation-related parameters.

Date	Key Events	INR
2025-11-17	Admission	1.55
2025-11-24	Emergency surgery; intraoperative IABP/ECMO placement; delayed sternal closure	1.65
2025-11-26	High chest tube output, wound oozing; bedside debridement and hemostasis	1.52
2025-11-27	Heparin initiated	1.30
2025-11-29	Warfarin initiated with heparin bridging	1.20
2025-11-30	Abdominal CT revealed intestinal obstruction; IABP balloon compressed the origin of the superior mesenteric artery; IABP removed	1.21
2025-12-02	Anticoagulation suspended; emergency exploratory laparotomy; extensive small bowel necrosis and mesenteric arteriovenous thrombosis	1.51
2025-12-03	Septic shock; death	1.45

## Discussion

BD is a chronic, relapsing systemic vasculitis of uncertain etiology. Its clinical features include recurrent oral and genital ulcers, uveitis, and skin lesions. The disease may affect multiple organ systems, such as blood vessels, heart, joints, lungs, nervous system, and gastrointestinal tract (8). In 1990, the International Study Group first published classification and diagnostic criteria for BD (9). These criteria were revised in 2014 to form the International Criteria for BD (ICBD), improving diagnostic sensitivity by including neurological and cardiovascular manifestations (10). The patient in this case met the ICBD criteria with a total score of 4 points (recurrent oral ulcers: 2 points; folliculitis-like skin lesions: 1 point; vascular involvement: 1 point), meeting the diagnostic threshold of ≥4 points. The clinical course and pathological evidence support a diagnosis of vascular BD. This provides critical pathological insight into the mechanism of thrombotic complications in this case.

In vascular BD, inflammation of the vascular wall is the primary driver of thrombosis, rather than a primary abnormality of the coagulation system (11). Inflammation drives thrombosis by inducing endothelial dysfunction, promoting platelet activation, and increasing tissue factor expression (4). At the same time, components of the coagulation system, such as fibrinogen, thrombin, factor Xa and factor VIIa, can influence immune cell function by binding to specific receptors, thereby amplifying the inflammatory response (12, 13). The pathological essence of vascular BD is inflammatory injury to the vessel wall rather than atherosclerosis or primary coagulation abnormalities. The inflammatory reaction is characterized predominantly by infiltration of neutrophils and T lymphocytes, involving the full thickness of the vessel wall, yet it primarily manifests as perivasculitis rather than the transmural necrotizing inflammation seen in classic systemic vasculitides (e.g., polyarteritis nodosa) (5). Vascular involvement is a major clinical manifestation of BD, presenting primarily as aneurysm formation or venous thrombotic occlusion (2, 14). Venous involvement is more common, accounting for over 80% of vascular BD cases, with superficial vein thrombosis and deep vein thrombosis being the most frequent presentations (15). The extensive mesenteric venous thrombosis in this case, which differs from IABP/ECMO-related arterial mesenteric ischemia (such as embolism or nonocclusive mesenteric ischemia), highlights the unique role of the venous system in thrombotic events in BD. On the basis of BD vasculitis, multiple risk factors—including IE, cardiac surgical trauma, mechanical circulatory support, and anatomical susceptibility—combined to ultimately result in catastrophic mesenteric ischemia.

The core mechanism of thrombosis in BD is vascular wall inflammation rather than a conventional hypercoagulable state; therefore, suppressing the inflammatory response is fundamental to preventing and treating thrombotic recurrence. In light of this, several recent guidelines have maintained a high degree of consensus: the 2018 EULAR guidelines (16) state that acute deep vein thrombosis should be managed primarily with immunosuppressants (e.g., glucocorticoids combined with azathioprine or cyclophosphamide) rather than anticoagulation alone; if anticoagulation is used, it must be combined with immunosuppressants and strictly exclude arterial aneurysms or active bleeding risk. For patients with arterial aneurysms, immunosuppression remains the cornerstone; in life-threatening situations, surgery or interventional procedures may be considered, but adequate perioperative immunosuppressive coverage is required. The 2025 EULAR guidelines (17) further clarify the priority of monoclonal TNF inhibitors in patients with vascular involvement, favoring infliximab over conventional immunosuppressants such as cyclophosphamide. Cardiac valvular involvement in BD closely mimics IE in clinical presentation, which can easily lead to misdiagnosis. In our patient, persistently negative blood cultures further complicated the differential diagnosis. Therefore, for BD patients with suspected IE and negative blood cultures, the differential diagnosis should integrate blood culture results with the typical features of BD, and the definitive diagnosis often relies heavily on histopathological evidence obtained from surgical specimens. If pathological examination reveals no evidence of infectious lesions, aseptic valvulitis should be strongly suspected, and immunosuppressive therapy should be the cornerstone of treatment; if pathological examination confirms IE, a balance between anti-infective and immunosuppressive therapies is required, along with timely surgical intervention and multidisciplinary management. The final diagnosis in this case was BD vasculitis complicated by IE, and the coexistence of both conditions significantly increased the complexity of diagnosis and treatment. Intraoperative findings of destructive valvular lesions and vegetations, combined with postoperative histopathological evidence, supported the diagnosis of IE; meanwhile, BD vasculitis accounted for the patient's underlying hypercoagulable state and vascular involvement. This dual diagnosis has direct implications for clinical management: a full course of anti-infective therapy is required for IE, while immunosuppression must be cautiously added on the basis of infection control to manage the underlying BD vasculitis—which also explains why anti-infective therapy alone was insufficient. In addition, the balance between anticoagulation and bleeding risk should be maintained throughout the entire treatment course, with close monitoring and dynamic adjustment.

## Conclusion

In this patient with vascular BD complicated by IE, catastrophic mesenteric ischemia—predominantly due to extensive mesenteric venous thrombosis—occurred during postoperative support with an IABP and ECMO following cardiac surgery, ultimately leading to intestinal necrosis, perforation, and death. This case suggests that, against a background of BD-related hypercoagulability, multiple factors—including cardiac surgical trauma, mechanical circulatory support, and delayed anticoagulation—may synergistically trigger a cascade amplification effect involving inflammation, hypercoagulability, and ischemia. For patients with concurrent IE, preoperative evaluation and active consideration of immunosuppressive and anti-infective therapy are warranted. During postoperative mechanical circulatory support, a high index of suspicion for mesenteric venous thrombosis should be maintained; if abdominal pain, a sharp rise in D-dimer, and a rapid increase in lactate develop, immediate abdominal CT assessment is indicated. In summary, extensive mesenteric venous thrombosis is a rare but fatal complication in patients with vascular BD and IE receiving postoperative mechanical circulatory support. The main limitation of this study is that it reports only a single fatal case, so causal conclusions cannot be made. Clinicians must fully recognize the interplay between BD-related hypercoagulability and the arterial factors associated with mechanical circulatory support, enhance early recognition and multidisciplinary collaboration, and thereby improve the prognosis of such patients.

## Data Availability

The original contributions presented in the study are included in the article/Supplementary Material, further inquiries can be directed to the corresponding author.
